# Metabolic Syndrome, and Particularly the Hypertriglyceridemic-Waist Phenotype, Increases Breast Cancer Risk, and Adiponectin Is a Potential Mechanism: A Case–Control Study in Chinese Women

**DOI:** 10.3389/fendo.2019.00905

**Published:** 2020-01-21

**Authors:** Yujuan Xiang, Wenzhong Zhou, Xuening Duan, Zhimin Fan, Shu Wang, Shuchen Liu, Liyuan Liu, Fei Wang, Lixiang Yu, Fei Zhou, Shuya Huang, Liang Li, Qiang Zhang, Qinye Fu, Zhongbing Ma, Dezong Gao, Shude Cui, Cuizhi Geng, Xuchen Cao, Zhenlin Yang, Xiang Wang, Hong Liang, Hongchuan Jiang, Haibo Wang, Guolou Li, Qitang Wang, Jianguo Zhang, Feng Jin, Jinhai Tang, Fuguo Tian, Chunmiao Ye, Zhigang Yu

**Affiliations:** ^1^Department of Breast Surgery, The Second Hospital of Shandong University, Jinan, China; ^2^Institute of Translational Medicine of Breast Disease Prevention and Treatment, Shandong University, Jinan, China; ^3^School of Medicine, Shandong University, Jinan, China; ^4^Breast Disease Center, Peking University First Hospital, Beijing, China; ^5^Department of Breast Surgery, The First Hospital of Jilin University, Changchun, China; ^6^Breast Disease Center, Peking University People's Hospital, Beijing, China; ^7^Department of Breast Surgery, Affiliated Tumor Hospital of Zhengzhou University, Zhengzhou, China; ^8^Breast Center, The Fourth Hospital of Hebei Medical University, Shijiazhuang, China; ^9^Department of Breast Surgery, Tianjin Medical University Cancer Institute and Hospital, Tianjin, China; ^10^Department of Thyroid and Breast Surgery, The First Affiliated Hospital of Binzhou Medical University, Binzhou, China; ^11^Department of Breast Surgery, Cancer Hospital, Chinese Academy of Medical Sciences, Beijing, China; ^12^Department of General Surgery, Linyi People's Hospital, Linyi, China; ^13^Department of General Surgery, Beijing Chaoyang Hospital, Beijing, China; ^14^Breast Center, Qingdao University Affiliated Hospital, Qingdao, China; ^15^Department of Breast and Thyroid Surgery, Weifang Traditional Chinese Hospital, Weifang, China; ^16^Department of Breast Surgery, The Second Affiliated Hospital of Qingdao Medical College, Qingdao Central Hospital, Qingdao, China; ^17^Department of General Surgery, The Second Affiliated Hospital of Harbin Medical University, Harbin, China; ^18^Department of Breast Surgery, The First Affiliated Hospital of China Medical University, Shenyang, China; ^19^Department of General Surgery, Nanjing Medical University Affiliated Cancer Hospital Cancer Institute of Jiangsu Province, Nanjing, China; ^20^Department of Breast Surgery, Shanxi Cancer Hospital, Taiyuan, China; ^21^Suzhou Institute, Shandong University, Suzhou, China

**Keywords:** breast cancer, metabolic syndrome, hypertriglyceridemic-waist phenotype, adiponectin, risk

## Abstract

**Objective:** To investigate the association between metabolic syndrome and breast cancer and to elucidate the potential mechanism underlying this association.

**Patients and Methods:** Based on baseline data drawn from 21 hospitals in 11 provinces of China, we performed a case–control study among 1,127 women (595 cases and 532 controls), divided into premenopausal, and postmenopausal subgroups. Student's *t* test, Pearson's χ^2^ test, and logistic regression analyses were performed to ascertain the association between breast cancer and metabolic syndrome, including all of its components. In addition, we attempted to clarify the potential role of adiponectin in this association.

**Results:** Among the components of metabolic syndrome, abnormal waist circumference was the component that markedly increased breast cancer risk in premenopausal women (OR 1.447, 95% CI 1.043–2.006). Metabolic syndrome with clusters of special risk factors showed an association with breast cancer risk. Among all these components of metabolic syndrome, the hypertriglyceridemic-waist (HW) phenotype significantly increased breast cancer risk (OR 1.56, 95% CI 1.02–2.39), regardless of menopausal status, rendering it a strong predictor of breast cancer. Total adiponectin levels and high-molecular-weight adiponectin were reversely associated with metabolic syndrome. In addition, total adiponectin levels among breast cancer patients were much lower than among controls (*p* = 0.005) only in the HW phenotype subgroup. Furthermore, the HW phenotype was associated with increased risk of estrogen receptor/progesterone receptor-positive (ER+/PR+) breast cancer, with a 95% (OR = 1.95, 95% CI:1.21–3.13) increase. However, there was no significant association between the HW phenotype and both ER+/PR– and ER–/PR– subtypes. These results suggested that low adiponectin levels may be a mechanism that explains the association between the HW phenotype and breast cancer risk.

**Conclusion:** Metabolic syndrome with special cluster factors is related to breast cancer risk; in particular, the HW phenotype can be regarded as a strong predictor of breast cancer. As an important factor involved in fat metabolism, adiponectin may strongly predict metabolic syndrome, especially the HW phenotype and breast cancer. Further research into this mechanism and epidemiological studies are needed. This study provides new evidence for the role of a healthy lifestyle in preventing breast cancer.

## Introduction

Breast cancer is known as the most prevalent cancer among women worldwide and has been the leading cause of female cancer deaths globally (1, 2). In China, breast cancer is also the most commonly diagnosed cancer and the sixth leading cause of cancer deaths among women, with an age-standardized rate (ASR) of 22.1 cases and 5.4 cases per 100,000 women, respectively, according to data from the GLOBOCAN 2012 (2, 3). In addition to the known risk factors associated with breast cancer, such as breastfeeding and number of childbirths, the westernization of traditional lifestyles has contributed substantially to this difference and is drawing more and more attention (4–6).

China has experienced fast economic growth and urbanization since the 1980s (7). Meanwhile, a rapid lifestyle transition has occurred, including nutrition changes characterized by increased energy intake from dietary fat and red meat, which increased, respectively, from 22 to 29.8% and from 9.3 to 13.7% between 1992 and 2002, and a sedentary lifestyle (7–9). Urbanization and the shift to a Westernized lifestyle have led to a substantial increase in a series of non-communicable chronic diseases, such as diabetes, obesity, metabolic syndrome, and cancers (10, 11). Currently, metabolic syndrome (MetS), a group of medical conditions that comprises obesity along with abnormal metabolic factors, including high blood pressure (BP), impaired fasting glucose (Glu), low high-density lipoprotein (HDL), and high triglycerides (TG) (12–15), represents one of the most complex public health challenges. Metabolic syndrome is defined as the coexistence of several risk factors for cardiovascular diseases, diabetes, and certain cancers, such as endometrial cancer, prostate cancer, colorectal cancer, and breast cancer (16).

Many studies have focused on abnormal metabolic factors separately from breast cancer risk (17). However, studies considering metabolic syndrome as an entity are comparatively scarce and the results of available studies have been inconsistent. Reports from most Western countries have confirmed the association in various subgroups (18–21), yet reports to the contrary do exist (22). A similar situation is present in studies among Asian populations (23–25). In China, there are much less epidemiological data on the relationship between metabolic syndrome as an entity and breast cancer risk (26). Moreover, as one of the components of metabolic syndrome, the hypertriglyceridemic-waist (HW) phenotype is characterized by the simultaneous presence of elevated waist circumference (WC) and concentration of triglycerides, which are strong predictors of chronic diseases, such as coronary artery disease, chronic kidney disease, and abnormal glucose metabolism (27, 28). Several studies have indicated that it is visceral obesity rather than subcutaneous obesity that relate to metabolic abnormalities (29). Therefore, the HW phenotype has emerged as a stronger predictor for those chronic diseases than metabolic syndrome, for it has been validated to be one of the convenient markers of visceral obesity (28). However, the relationship between this typical phenotype and breast cancer is still unclear. The Chinese population is more likely to be viscerally obese or centrally obese in spite of generally having a low BMI (29–31). For this reason, investigating the association between the HW phenotype and breast cancer is necessary and may provide new insight into the prevention of the disease.

Figuring out the mechanisms underlying how metabolic syndrome is associated with breast cancer is particular important, as the negative impact of these risk factors could be attenuated to some extent through lifestyle intervention and conservative therapy of underlying metabolic conditions. Insulin resistance and chronic inflammation have come to be regarded as the two main mechanisms bridging metabolic syndrome and breast cancer (32, 33). Adipokines, which are involved in both insulin resistance and chronic inflammation, have also been demonstrated to contribute to the pathogenesis of abnormal metabolic factors, including obesity, diabetes, hyperlipemia, and high BP (34, 35). The dysregulation of adiponectin, the most abundant adipokine (36), does not only play a role in metabolic syndrome but also in breast cancer (37). Our previous meta-analysis and epidemiological results confirmed that a higher circulating high-molecular-weight (HMW) adiponectin (known as the active form) decreased breast cancer risk, especially in postmenopausal women (38, 39). However, most reported results were obtained from cellular and molecular experiments, and there have been few studies with intact data systematically assessing adiponectin as the molecular mechanism underlying the association between metabolic syndrome and breast cancer morbidity.

Therefore, the aim of this study was to evaluate the association between metabolic syndrome and breast cancer risk in a large-scale sample of Chinese women at large scale and to research whether adiponectin could link metabolic syndrome and breast cancer as a potential molecular mechanism, to provide new insight into the prevention of the disease.

## Materials and Methods

### Patients and Public Involvement

A multicenter stratified inclusion process was used to enroll the participants from 21 hospitals in 11 provinces in northern and eastern China from April 2012 to April 2013. All participants were voluntarily involved in our research, including finishing a self-designed questionnaire that was previously developed to record information through person-to-person interviews. All trained staffs were involved in the recruitment of participants and conduct of the study. Written informed consent was obtained from each participant by investigators as part of the interview. The patients' advisors have been thanked in the Acknowledgments section.

### Study Participants

The inclusion criteria for cases were as follows: (1) newly diagnosed and histologically confirmed breast cancer; (2) Han ethnic group; and (3) aged 25–70 years old. For the control group, the following criteria were used: (1) negative physical examination results; (2) negative ultrasound breast scans and/or mammographic screening results; (3) matched age with cases (±3 years); (4) women who had been hospitalized or had a regular physical examination in the same hospital as matched case in the same time period; (5) no evidence of cancer or history of cancer; and (6) Han ethnic group. Furthermore, all included cases and controls should have complete data on metabolic factors, adiponectin levels by ELISA, and anthropometric measurements.

Upon applying the aforementioned criteria, there were a total 1,127 participants (595 cases and 532 controls) included in this study. According to menopausal status and excluding 5 participants with unknown menopausal status, 383 cases, and 339 controls were included in the premenopausal subgroup; 209 cases and 191 controls were included in the postmenopausal subgroup. The study protocols and procedures were approved by the Institutional Review Board at the Second Hospital of Shandong University.

### Data Collection

Data were obtained through in-person interviews based on a self-designed, structured questionnaire. The questionnaire contained six sections: (1) demographic characteristics and female physiological and reproductive factors; (2) medical and family history: primarily, breast-related diseases, and family history of breast cancer; (3) lifestyle habits; (4) medication and chemical exposure history; (5) breast cancer-related knowledge; and (6) medical records. The histological and immunohistochemical diagnoses of breast cancer patients were also collected from the medical records.

Anthropometric measurement was conducted by clinicians. WC was measured to the nearest 0.1 cm, with participants wearing light clothing. High BP was defined as ≥130 mmHg systolic BP or ≥85 mmHg diastolic BP or under antihypertensive drug treatment for patients with a history of hypertension, as it was nearly the same in the four criteria for metabolic syndrome (Supplemental Table 1).

### Laboratory Analyses

Total and HMW adiponectin levels were assayed from plasma using human total adiponectin and HMW adiponectin quantitative ELISA kits, respectively (SRP300, SHWAD0; RD Systems). Each sample was assayed twice and the average of the data was used. No samples were below the detection limits. All analyses were performed according to the manufacturer's recommended protocols.

### Definition of Metabolic Syndrome and HW Phenotype

The criteria for metabolic syndrome have been defined by four different organizations, including the National Cholesterol Education Program Adult Treatment Panel III (NCEP-ATP III) in the United States in 2005 (12), the International Diabetes Federation (IDF) in 2005 (12), the Chinese Diabetes Society (CDS) in 2007 (15), and the IDF and NCEP-ATPIII Joint Interim Statement published in 2009 (14) (Supplemental Table 1).

The HW phenotype was represented by the simultaneous presence of elevated WC (≥90 cm for men, ≥80 cm for women) and elevated serum triglyceride concentrations (TG concentrations ≥1.7 mmol/L) according to pre-determined cutoff points and criteria used in published work with Chinese populations (40, 41).

### Statistical Analysis

We analyzed and compared the distributions of metabolic syndrome, components, and adiponectin in the case and control groups. Descriptive characteristic variables were expressed as means ± standard deviations (SD). *P*-values of continuous variables were determined by Student *t* tests and those of categorical variables by chi-squared tests. Odds ratios (ORs) and 95% confidence intervals (CIs) were obtained by single binary logistic regression analyses, and factors were related to breast cancer. Covariates considered in the adjusted model included age, number of childbirths (≤1, 2, ≥3), age at menarche (≤13, 14, ≥15 years), breastfeeding (yes, no), smoking (never, occasionally, regularly), alcohol use (never, occasionally, regularly), family history of breast cancer (yes, no), and contraceptive drug use (yes, no).

All *P*-values were two-sided and *P* < 0.05 was considered significant. All analyses were performed using IBM SPSS 21.0 statistical software (IBM Corp., Armonk, NY, USA).

## Results

### Participant Characteristics

The demographic and clinical characteristics of the participants are shown in Table 1. Education level and number of births showed a significant difference between cases and controls (*P* < 0.001). A family history of breast cancer was more prevalent among cases than controls. Other factors, such as breastfeeding and age at menarche, showed no differences. Among metabolic components in metabolic syndrome, WC was greater among cases; other metabolic factors showed no statistical significance.

**Table 1:** Participant characteristics.

	**All subjects**		
	**Case (*n* = 595)**	**Control (*n* = 532)**	***P***
	**Mean ± SD or *n* (%)**	**Mean ± SD or *n* (%)**	
Age	48.13 ± 8.33	47.67 ± 8.50	0.365
Education			<0.001
Primary school or below	112 (19.3%)	69 (13.3%)	
Junior school	200 (34.5%)	135 (26.1%)	
Senior school	183 (31.6%)	178 (34.4%)	
Junior college or above	85 (14.6%)	136 (26.2%)	
n/a	29		
Number of births			<0.001
≤1	301 (51.1%)	350 (66.2%)	
2	213 (36.2%)	136 (25.7%)	
≥3	75 (12.7%)	43 (8.1%)	
n/a	9		
Age at menarche			0.452
≤13	141 (24.1%)	136 (26.0%)	
14	170 (29.1%)	135 (25.8%)	
≥15	273 (46.7%)	252 (48.2%)	
n/a	20		
Menopause status		0.798	
Yes	209 (35.3%)	191 (36.0%)	
No	383 (64.7%)	339 (64.0%)	
n/a	5		
Breastfeeding		0.083	
Yes	522 (91.7%)	491 (94.4%)	
No	47 (8.3%)	29 (5.6%)	
n/a	38		
Contraceptive drugs		0.422	
Yes	52 (9.1%)	40 (7.7%)	
No	520 (90.9%)	477 (92.3%)	
n/a	38		
Family history		0.023	
Yes	42 (7.4%)	21 (4.1%)	
No	526 (92.6%)	486 (95.9%)	
n/a	52		
Alcohol consumption		0.335	
Never	515 (87.6%)	475 (90.1%)	
Occasionally	67 (11.4%)	46 (8.7%)	
Constantly	6 (1.0%)	6 (1.1%)	
n/a	12		
Smoking		0.420	
Never	564 (95.9%)	564 (95.9%)	
Occasionally	7 (1.2%)	7 (1.2%)	
Constantly	17 (2.9%)	17 (2.9%)	
n/a	11		
Waist circumference	80.81 ± 9.77	78.78 ± 8.07	<0.001
Fasting serum glucose	5.30 ± 1.05	5.26 ± 1.20	0.638
Triglycerides	1.23 ± 0.83	1.28 ± 0.80	0.317
High-density lipoprotein	1.58 ± 0.58	1.53 ± 0.54	0.090
High blood pressure		0.147	
Yes	256 (43.6%)	204 (39.3%)	
No	331 (56.4%)	315 (60.7%)	
n/a	21		

### IDF Criteria Are Superior for Evaluating the Risk

With greater understanding of metabolic syndrome, the corresponding criteria have changed. To more reasonably assess the influence of metabolic syndrome on breast cancer risk and determine the most appropriate subject of further investigation, we compared four diagnostic criteria. The results (Table 2) confirmed that the 2005 definition of the IDF yielded only positive results, with OR 1.530 (95% CI 1.074–2.197). Moreover, by IDF criteria, the result of the *P*_trend_ indicated that breast cancer morbidity increased with the number of abnormal factors in each cluster.

**Table 2:** Aggregation of metabolic factors and breast cancer.

**Diagnostic criteria**	**Aggregation of metabolic factors**	**All subjects**			
		**Case**	**Control**	**OR**	**95% CI**
IDF 2005	0	312 (52.4%)	325 (61.1%)	1.000	
	1–2	131 (22.0%)	90 (16.9%)	1.516	1.112–2.067
	3	94 (15.8%)	64 (12.0%)	1.530	1.074–2.179
	4	32 (5.4%)	39 (7.3%)	0.855	0.522–1.399
	5	26 (4.4%)	14 (2.6%)	1.935	0.992–3.773
	*P*_trend_			0.039	
ATPIII 2005	0	109 (18.3%)	99 (18.6%)	1.000	
	1–2	331 (55.6%)	284 (53.4%)	1.059	0.773–1.450
	3	95 (16.0%)	102 (19.2%)	0.846	0.573–1.249
	4	41 (6.9%)	36 (6.8%)	1.034	0.613–1.747
	5	19 (3.2%)	11 (2.1%)	1.569	0.711–3.460
	*P*_trend_			0.514	
CDS 2007	0	202 (33.9%)	169 (31.8%)	1.000	
	1–2	321 (53.9%)	309 (58.1%)	0.869	0.672–1.124
	3	54 (9.1%)	43 (8.1%)	1.051	0.670–1.647
	4	13 (2.2%)	9 (1.7%)	1.208	0.504–2.896
	5	5 (0.8%)	2 (0.4%)	2.092	0.401–10.918
	*P*_trend_			0.781	
Joint statement 2009	0	109 (18.3%)	99 (18.6%)	1.000	
	1–2	331 (55.6%)	284 (53.4%)	1.059	0.773–1.450
	3	95 (16.0%)	102 (19.2%)	0.846	0.573–1.249
	4	41 (6.9%)	36 (6.8%)	1.034	0.613–1.747
	5	19 (3.2%)	11 (2.1%)	1.569	0.711–3.460
	*P*_trend_			0.514	

### As a Metabolic Symptom Component, WC Increases Breast Cancer Risk

Using IDF criteria, we were able to categorize the variables and analyze the associations between breast cancer and metabolic factors according to menopausal status. By synthesizing the results (Tables 3, 4), we found that WC (an indicator of abdominal obesity) was associated with breast cancer risk in the premenopausal subgroup. Moreover, by conducting a multivariate logistic regression, a larger WC significantly increased breast cancer risk, with OR 1.447 (95% CI 1.043–2.006). However, other metabolic factors showed no association with breast cancer risk.

**Table 3:** Association between metabolic syndrome components and breast cancer by chi-squared test.

	**All subjects**			**Premenopausal**			**Postmenopausal**		
	**Case**	**Control**	***P***	**Case**	**Control**	***P***	**Case**	**Control**	***P***
Waist circumference			0.003			0.010			0.104
≥80 cm	283 (47.6%)	207 (38.9%)		161 (42.0%)	111 (32.7%)		122 (58.4%)	96 (50.3%)	
<80 cm	312 (52.4%)	325 (61.1%)		222 (58.0%)	228 (67.3%)		87 (41.6%)	95 (49.7%)	
n/a	0			0			0		
Fasting serum glucose			0.186			0.133			0.734
≥5.6 mmol/L	164 (27.6%)	126 (24.1%)		84 (21.9%)	59 (17.5%)		80 (38.3%)	67 (36.6%)	
<5.6 mmol/L	431 (72.4%)	397 (75.9%)		299 (78.1%)	279 (82.5%)		129 (61.7%)	116 (63.4%)	
n/a	9			1			8		
Triglycerides			0.142			0.435			0.247
≥1.7 mmol/L	101 (17.3%)	108 (20.7%)		46 (12.1%)	47 (14.1%)		55 (27.1%)	61 (32.4%)	
<1.7 mmol/L	484 (82.7%)	413 (79.3%)		333 (87.9%)	286 (85.9%)		148 (72.9%)	127 (67.6%)	
n/a	21			10			9		
High-density lipoprotein		0.702			0.643			0.877	
<1.29 mmol/L	63 (10.6%)	51 (9.9%)		44 (11.5%)	34 (10.4%)		18 (8.7%)	17 (9.1%)	
≥1.29 mmol/L	530 (89.4%)	463 (90.1%)		339 (88.5%)	293 (89.6%)		189 (91.3%)	169 (90.9%)	
n/a	20			12			7		
High blood pressure			0.147			0.294			0.262
Yes	256 (43.6%)	204 (39.3%)		256 (43.6%)	204 (39.3%)		124 (59.9%)	101 (54.3%)	
No	331 (56.4%)	315 (60.7%)		331 (56.4%)	315 (60.7%)		83 (40.1%)	85 (45.7%)	
n/a	21			14			7		

**Table 4:** Association between metabolic symptom components and breast cancer by multivariate logistic regression.

	**All subjects**			**Premenopausal**			**Postmenopausal**		
	**OR**	**95% CI**	***P***	**OR**	**95% CI**	***P***	**OR**	**95% CI**	***P***
**Multivariate model**^**a**^									
Fasting serum glucose (normal = reference)	1.144	0.852–1.536	0.371	1.371	0.916–2.052	0.125	1.010	0.629–1.622	0.966
Waist circumference (normal = reference)	1.344	1.039–1.739	0.024	1.447	1.043–2.006	0.027	1.478	0.937–2.331	0.093
High-density lipoprotein (normal = reference)	0.927	0.608–1.415	0.726	1.064	0.644–1.756	0.809	0.600	0.262–1.378	0.229
Triglycerides (normal = reference)	0.798	0.590–1.079	0.142	0.751	0.471–1.198	0.230	0.634	0.387–1.050	0.077
High blood pressure (no = reference)	1.091	0.834–1.427	0.526	1.036	0.736–1.460	0.839	1.359	0.854–2.161	0.195

a *Adjusted for age, number of childbirths, age at menarche, breastfeeding, smoking, alcohol use, family history of breast cancer, and contraceptive drug use*.

### Cluster Mode of HW Phenotype Significantly Increases Breast Cancer Risk

The different aggregation patterns that met the diagnosis were analyzed. As shown in Table 5, women with premenopausal metabolic syndrome who had abnormal values for WC+HDL+TG showed the highest breast cancer risk. As for the postmenopausal group, a greater number of abnormal conditions including WC+Glu+TG, WC+Glu+HDL+TG, WC+HDL+TG+BP, and WC+Glu+TG+BP increased breast cancer risk, with clusters of nearly all four risk factors. In postmenopausal women, abnormal WC+BP+TG was borderline significantly related to breast cancer risk.

**Table 5:** Aggregation factors in metabolic syndrome and breast cancer risk, by IDF criteria.

	**All subjects**			**Premenopausal**			**Postmenopausal**		
	**Case (*n* = 595)**	**Control (*n* = 532)**	***P***	**Case (*n* = 383)**	**Control (*n* = 339)**	***P***	**Case (*n* = 209)**	**Control (*n* = 191)**	***P***
WC+Glu+HDL			0.017			0.124			0.060
Yes	46 (7.7%)	23 (4.3%)		23 (6.0%)	12 (3.5%)		23 (11.0%)	11 (5.8%)	
No	549 (92.3%)	509 (95.7%)		360 (94.0%)	327 (96.5%)		186 (89.0%)	180 (94.2%)	
WC+Glu+BP			0.131			0.532			0.107
Yes	51 (8.6%)	33 (6.2%)		27 (7.0%)	20 (5.9%)		24 (11.5%)	13 (6.8%)	
No	544 (91.4%)	499 (93.8%)		356 (93.0%)	319 (94.1%)		185 (88.5%)	178 (93.2%)	
WC+Glu+TG			0.009			0.092			0.037
Yes	33 (5.5%)	13 (2.4%)		18 (4.7%)	8 (2.4%)		15 (7.2%)	5 (2.6%)	
No	562 (94.5%)	519 (97.6%)		365 (95.3%)	331 (97.6%)		194 (92.8%)	186 (97.4%)	
WC+HDL+BP			0.043			0.162			0.130
Yes	63 (10.6%)	38 (7.1%)		33 (8.6%)	20 (5.9%)		30 (14.4%)	18 (9.4%)	
No	532 (89.4%)	494 (92.9%)		350 (91.4%)	319 (94.1%)		179 (85.6%)	173 (90.6%)	
WC+HDL+TG			0.007			0.043			0.073
Yes	43 (7.2%)	19 (3.6%)		25 (6.5%)	11 (3.2%)		18 (8.6%)	8 (4.2%)	
No	552 (92.8%)	513 (96.4%)		358 (93.5%)	328 (96.8%)		191 (91.4%)	183 (95.8%)	
WC+BP+TG			0.094			0.619			0.051
Yes	42 (91.4%)	25 (91.4%)		20 (5.2%)	15 (4.4%)		22 (10.5%)	10 (5.2%)	
No	553 (91.4%)	507 (91.4%)		363 (94.8%)	324 (95.6%)		187 (89.5%)	181 (94.8%)	
WC+Glu+HDL+BP			0.331			0.971			0.148
Yes	23 (3.9%)	15 (2.8%)		10 (2.6%)	9 (2.7%)		13 (6.2%)	6 (3.1%)	
No	572 (96.1%)	517 (97.2%)		373 (97.4%)	330 (97.3%)		196 (93.8%)	185 (96.9%)	
WC+Glu+HDL+TG			0.007			0.068			0.045
Yes	25 (4.2%)	8 (1.5%)		14 (3.7%)	5 (1.5%)		11 (5.3%)	3 (1.6%)	
No	570 (95.8%)	524 (98.5%)		369 (96.3%)	334 (98.5%)		198 (94.7%)	188 (98.4%)	
WC+HDL+TG+BP			0.022			0.204			0.041
Yes	27 (4.5%)	11 (2.1%)		14 (3.7%)	7 (2.1%)		13 (6.2%)	4 (2.1%)	
No	568 (95.5%)	521 (97.9%)		369 (96.3%)	332 (97.9%)		196 (93.8%)	187 (97.9%)	
WC+Glu+TG+BP			0.022			0.285			0.028
Yes	22 (3.7%)	8 (1.5%)		10 (2.6%)	5 (1.5%)		12 (5.7%)	3 (1.6%)	
No	573 (96.3%)	524 (98.5%)		373 (97.4%)	334 (98.5%)		197 (94.3%)	188 (98.4%)	
All factors			0.059			0.340			0.075
Yes	16 (2.7%)	6 (1.1%)		8 (2.1%)	4 (1.2%)		8 (3.8%)	2 (1.0%)	
No	579 (97.3%)	526 (98.9%)		375 (97.9%)	335 (98.8%)		201 (96.2%)	189 (99.0%)	

*WC, waist circumference; Glu, fasting serum glucose; BP, blood pressure; TG, triglycerides; HDL, high-density lipoprotein*.

Abnormal WC+TG, known as HW phenotype, can be seen in all positive results in Table 6, in all participants and subgroups. By conducting a logistic regression, it was evident that the HW phenotype significantly increased breast cancer risk, with an OR 1.563 (95% CI 1.023–2.387), regardless of menopausal status (Table 6). Although not significant in both pre- and postmenopausal subgroups, OR values (1.492 and 1.599, respectively) predicted a link with breast cancer to some degree.

**Table 6:** Association between HW phenotype and breast cancer by logistic regression.

	**All subjects(*****n*** **=** **595)**			**Premenopausal(*****n*** **=** **383)**			**Postmenopausal(*****n*** **=** **209)**			**ER+/PR+(*****n*** **=** **293)**			**ER+/PR–(*****n*** **=** **59)**			**ER–/PR–(*****n*** **=** **148)**		
	**OR**	**95% CI**	***P***	**OR**	**95% CI**	***P***	**OR**	**95% CI**	***P***	**OR**	**95% CI**	***P***	**OR**	**95% CI**	***P***	**OR**	**95% CI**	***P***
**Univariate model**																		
WC+TG (normal = reference)	1.66	1.10–2.50	0.016	1.63	0.95–2.82	0.077	1.72	0.91–3.22	0.09	2.06	1.29–3.27	0.002	1.70	0.73–4.00	0.222	1.43	0.76–2.67	0.266
**Multivariate model^a^**																		
WC+TG (normal=reference)	1.56	1.02–2.39	0.039	1.49	0.85–2.63	0.167	1.60	0.82–3.12	0.170	1.95	1.21–3.13	0.006	1.71	0.72–4.08	0.225	1.21	0.63–2.33	0.571

*WC, waist circumference; TG, triglycerides; ER, estrogen receptor; PR, progesterone receptor*.

a *Adjusted for age, number of childbirths, age at menarche, breastfeeding, smoking, alcohol use, family history of breast cancer, and contraceptive drug use*.

We also investigated associations between HW phenotype and breast cancer risk according to joint ER/PR status. Similar to what was found in all participant, HW phenotype was associated with ER+/PR+ breast cancer, with a 95% (OR = 1.95, 95% CI:1.21–3.13) increase in risk for women with a positive HW phenotype. However, there was no significant association between HW phenotype and both ER+/PR– and ER–/PR– subtypes.

### Adiponectin Might Be the Mechanism Linking Metabolic Syndrome to Breast Cancer

As a latent mechanism, the effects of adiponectin on metabolic syndrome warrant investigation. As shown in Table 7, total adiponectin and HMW adiponectin were reversely associated with metabolic syndrome regardless of menopausal status. Similar results were obtained for total adiponectin with WC and TG, and HMW adiponectin with TG (Table 8). Differing by subgroup, total adiponectin and HMW adiponectin were associated with Glu in premenopausal women but with HDL in postmenopausal women (Table 8). Nevertheless, the HMW/total ratio was not correlated with metabolic syndrome and any of its components.

**Table 7:** Association between total adiponectin, HMW adiponectin, HMW/total ratio, and metabolic syndrome.

	**All subjects**			**Premenopausal**			**Postmenopausal**		
	**With MetS**	**Without MetS**	***p***	**With MetS**	**Without MetS**	***p***	**With MetS**	**Without MetS**	***p***
Total adiponectin	5.970 ± 3.789	2.807 ± 2.007	0.004	5.960 ± 3.830	6.637 ± 3.558	0.054	5.979 ± 3.762	6.909 ± 3.875	0.022
HMW adiponectin	2.408 ± 1.870	2.807 ± 2.007	0.004	2.371 ± 1.830	2.757 ± 1.958	0.037	2.445 ± 1.915	2.935 ± 2.116	0.024
HMW/total ratio	0.39 ± 0.14	0.41 ± 0.16	0.101	0.39 ± 0.14	0.40 ± 0.17	0.233	0.39 ± 0.15	0.42 ± 0.15	0.150

*MetS, metabolic syndrome; HMW, high molecular weight*.

**Table 8:** Association between total adiponectin, HMW adiponectin, HMW/total ratio, and all components of metabolic syndrome.

	**Waist circumference (cm)**			**Fasting serum glucose (mmol/L)**			**Triglycerides (mmol/L)**			**HDL (mmol/L)**		**High blood pressure**			
	**≥80**	** <80**	***P***	**≥5.6**	** <5.6**	***P***	**≥1.7**	** <1.7**	***P***	** <1.29**	**≥1.29**	***P***	**Yes**	**No**	***P***
**ALL SUBJECTS**															
Total adiponectin	6.135 ± 3.569	6.832 ± 3.775	0.002	5.981 ± 3.649	6.706 ± 3.690	0.004	5.26 ± 2.957	6.762 ± 3.823	<0.001	5.555 ± 3.410	6.641 ± 3.703	0.003	6.504 ± 3.723	6.553 ± 3.680	0.829
HMW adiponectin	2.648 ± 2.057	2.761 ± 1.923	0.343	2.484 ± 1.909	2.783 ± 1.998	0.027	2.264 ± 1.688	2.802 ± 2.019	<0.001	2.239 ± 1.648	2.765 ± 2.009	0.007	2.826 ± 2.106	2.620 ± 1.65	0.087
HMW/total ratio	0.41 ± 0.15	0.40 ± 0.16	0.107	0.40 ± 0.15	0.40 ± 0.16	0.447	0.39 ± 0.15	0.40 ± 0.16	0.136	0.38 ± 0.15	0.40 ± 0.16	0.177	0.42 ± 0.15	0.39 ± 0.16	0.004
**PREMENOPAUSAL**															
Total adiponectin	6.151 ± 3.569	6.716 ± 3.632	0.041	5.863 ± 3.704	6.665 ± 3.582	0.017	5.508 ± 2.917	6.655 ± 3.703	0.004	5.782 ± 3.748	6.5.0 ± 3.566	0.061	6.378 ± 3.492	6.559 ± 3.674	0.534
HMW adiponectin	2.658 ± 2.013	2.702 ± 1.896	0.767	2.398 ± 1.894	2.759 ± 1.947	0.047	2.163 ± 1.620	2.758 ± 1.979	0.006	2.333 ± 1.750	2.729 ± 1.958	0.089	2.737 ± 1.886	2.642 ± 1.939	0.540
HMW/total ratio	0.41 ± 0.15	0.39 ± 0.17	0.133	0.39 ± 0.15	0.40 ± 0.16	0.357	0.37 ± 0.15	0.40 ± 0.16	0.050	0.38 ± 0.16	0.40 ± 0.16	0.348	0.42 ± 0.15	0.39 ± 0.16	0.024
**POSTMENOPAUSAL**															
Total adiponectin	6.115 ± 3.578	7.171 ± 41.05	0.007	6.096 ± 3.603	6.837 ± 3.943	0.064	5.541 ± 3.001	7.023 ± 4.075	<0.001	4.924 ± 2.396	6.766 ± 3.933	<0.001	6.667 ± 3.952	6.537 ± 3.707	0.056
HMW adiponectin	2.635 ± 2.116	2.931 ± 1.988	0.154	2.568 ± 1.926	2.859 ± 2.120	0.174	2.344 ± 1.743	2.908 ± 2.110	0.012	1.965 ± 1.357	2.846 ± 2.097	0.001	2.938 ± 2.316	2.555 ± 1.639	0.742
HMW/total ratio	0.41 ± 0.16	0.40 ± 0.14	0.665	0.40 ± 0.15	0.41 ± 0.15	0.648	0.40 ± 0.15	0.41 ± 0.15	0.734	0.38 ± 0.15	0.41 ± 0.15	0.276	0.42 ± 0.15	0.39 ± 0.15	0.097

*HDL, high-density lipoprotein*.

In addition, we proceeded to analyze the possible association among breast cancer, metabolic syndrome, and adiponectin. We found that such a relationship indeed existed, for there was significant difference in total adiponectin levels between breast cancer patients and the controls only in the population with the HW phenotype. As shown in Table 9, total adiponectin levels among breast cancer patients were much lower than among the controls (*p* = 0.005) in the HW phenotype subgroup.

**Table 9:** The association among metabolic syndrome, breast cancer, and adiponectin.

		**Controls**	**All cases**		**ER+/PR+**		**ER+/PR–**		**ER–/PR–**	
**METABOLIC SYNDROME**										
YES										
	Total adiponectin			0.362		0.944		0.764		0.203
	High	26 (22.2%)	27 (17.8%)		17 (21.8%)		3 (15.8%)		5 (12.8%)	
	Low	91 (77.8%)	125 (82.2%)		61 (78.2%)		16 (84.2%)		34 (87.2%)	
	HMW adiponectin			0.296		0.597		0.113		0.403
	High	66 (56.4%)	76 (50.0%)		41 (52.6%)		7 (36.8%)		19 (48.7%)	
	Low	51 (43.6%)	76 (50.0%)		37 (47.4%)		12 (63.2%)		20 (51.3%)	
	HMW/total ratio			0.354		0.069		0.805		0.711
	High	59 (50.4%)	68 (44.7%)		29 (37.2%)		9 (47.4%)		21 (53.8%)	
	Low	58 (49.6%)	84 (55.3%)		49 (62.8%)		10 (52.6%)		18 (46.2%)	
No										
	Total adiponectin			0.097		0.121		0.339		0.118
	High	106 (25.5%)	92 (20.8%)		43 (20.0%)		13 (32.5%)		20 (18.3%)	
	Low	309 (74.5%)	351 (79.2%)		172 (80.0%)		27 (67.5%)		89 (81.7%)	
	HMW adiponectin			0.507		0.970		0.588		0.244
	High	287 (69.2%)	297 (67.0%)		149 (69.3%)		26 (65.0%)		69 (63.3%)	
	Low	128 (30.8%)	146 (33.0%)		66 (30.7%)		14 (35.0%)		40 (36.7%)	
	HMW/total ratio			0.359		0.229		0.873		0.062
	High	213 (51.3%)	213 (48.2%)		99 (46.3%)		20 (50.0%)		45 (41.3%)	
	Low	202 (48.7%)	229 (51.8%)		115 (53.7%)		20 (50.0%)		64 (58.7%)	
**HW PHENOTYPE**										
YES										
	Total adiponectin			0.005		0.028		1.000		0.043
	High	14 (35.9%)	9 (13.0%)		6 (14.6%)		2 (28.6%)		1 (6.7%)	
	Low	25 (64.1%)	60 (87.0%)		35 (85.4%)		5 (71.4%)		14 (93.3%)	
	HMW adiponectin			0.717		0.527		0.424		0.583
	High	24 (61.5%)	40 (58.0%)		28 (68.3%)		3 (42.9%)		8 (53.3%)	
	Low	15 (38.5%)	29 (42.0%)		13 (31.7%)		4 (57.1%)		7 (46.7%)	
	HMW/total ratio			0.570		0.263		1.000		0.839
	High	17 (43.6%)	34 (49.3%)		23 (56.1%)		3 (42.9%)		7 (46.7%)	
	Low	22 (56.4%)	35 (50.7%)		18 (43.9%)		4 (57.1%)		8 (53.3%)	
NO										
	Total adiponectin			0.247		0.442		0.632		0.150
	High	118 (23.9%)	110 (20.9%)		54 (21.4%)		14 (26.9%)		24 (18.0%)	
	Low	375 (76.1%)	416 (79.1%)		198 (78.6%)		38 (73.1%)		109 (82.0%)	
	HMW adiponectin			0.252		0.505		0.191		0.157
	High	329 (66.7%)	333 (63.3%)		162 (64.3%)		30 (57.7%)		80 (60.2%)	
	Low	164 (33.3%)	193 (36.7%)		90 (35.7%)		22 (42.3%)		53 (39.8%)	
	HMW/total ratio			0.136		0.011		0.813		0.132
	High	255 (51.7%)	247 (47.0%)		105 (41.8%)		26 (50.0%)		59 (44.4%)	
	Low	238 (48.3%)	278 (53.0%)		146 (58.2%)		26 (50.0%)		74 (55.6%)	

*ER, estrogen receptor; PR, progesterone receptor*.

*Cut-off value of high and low level for total adiponectin, HMW adiponectin, and HMW/total ratio is 8.768, 1.635, and 0.399, respectively*.

To clarify the role of adiponectin in breast cancer depending on hormone receptor, we also conducted a subgroup analysis by joint ER/PR status. Similar to the findings regarding the association between the HW phenotype and breast cancer risk, there was a significant difference of total adiponectin in ER+/PR+ (*p* = 0.028) and ER–/PR– (*p* = 0.043) breast cancer compared to the controls, who were much lower in the HW phenotype subgroup. Conversely, such a difference was not found in women without the HW phenotype as well as ER+/PR– breast cancer with the HW phenotype.

## Discussion

In this case–control study, we found that among components in metabolic syndrome by IDF criteria, WC (an indicator of central obesity) was strongly associated with increased premenopausal breast cancer risk. Metabolic syndrome, especially the clustering of three or four components, increases breast cancer risk, which is more common in postmenopausal women. Across various aggregation patterns, the HW phenotype showed a strong correlation with increased breast cancer risk.

Metabolic syndrome, composed of five aberrant metabolic factors, is receiving growing attention because of its close link with lifestyle (12). There are only few epidemiological studies in this area worldwide, which have yielded conflicting results. In addition to differences in study design, sample size, and ethnic groups studied, the diagnostic criteria adopted for metabolic syndrome might have contributed to the conflicting results. Therefore, we compared the four most recent metabolic syndrome definitions and found the 2005 IDF definition to be the most appropriate. This is the first time that the diagnostic criteria of the 2009 Joint Statement have been used to evaluate the influence of metabolic syndrome on breast cancer risk. Consistent with our results, a study in Korea also compared two sets of diagnostic criteria for metabolic syndrome and confirmed that the IDF criteria were superior (23). In assessing breast cancer risk, Rodriguez-Ortiz et al. (37) used IDF and ATP criteria to evaluate metabolic syndrome remission in a cohort of patients undergoing Roux-en-Y gastric bypass; the IDF criteria were confirmed as more suitable, which further verified that different metabolic syndrome criteria might yield correspondingly discrepant results.

In the present study, among components of metabolic syndrome, increased WC was the only factor related to increased premenopausal breast cancer risk. Although it is generally known that obesity is positively associated with postmenopausal breast cancer risk, this association in premenopausal women remains controversial (42–44). Central obesity, which reflects visceral fat as measured by WC or waist-to-hip ratio, could more accurately explain obesity-related health risk (45) than body mass index (BMI), which is a measure of both adipose tissue and lean mass (46). In line with our study, Nagrani et al. observed that a larger WC was associated with a threefold increased risk of breast cancer regardless of menopausal status (47). Moreover, a dose–response meta-analysis of prospective studies also confirmed that central obesity as measured by WC was associated with increased risk of premenopausal breast cancer after adjustment for BMI (48).

To thoroughly evaluate the effect of metabolic syndrome on breast cancer risk, it is important to assess the influence on breast cancer risk exerted by cluster patterns among various metabolic factors, which in the last decade has only been investigated in a study by Wang et al. (26). Correspondingly, in our study, various aggregations gave rise to different effects. Furthermore, by crossing positive associations of cluster patterns with cancer risk, abnormal WC+TG had a fundamental predictive influence on this association. Defined as HW phenotype, its involvement in coronary artery disease, insulin resistance, and hypertension has been confirmed (49–51). As WC cannot fully discriminate visceral adiposity from subcutaneous abdominal adiposity, elevated triglyceride (TG) levels have been adopted as a marker of dysfunctional visceral adipose tissue. Published data have demonstrated that the HW phenotype is a stronger predictor of certain chronic diseases than WC, BMI, as well as metabolic syndrome. As the Chinese population is likely to be viscerally obese or centrally obese in spite of generally having a low BMI, we should make efforts to improve the metabolic health of this high-risk group, and encourage their engagement in early intensive lifestyle modification, as simple weight loss might not be the optimal solution for them. Notably, to our knowledge, our study is the first to evaluate the role of the HW phenotype in breast cancer risk, even in all cancers. Verified increased inflammation in this phenotype may provide an explanation for this increased risk (52).

In terms of the mechanisms underlying the association between metabolic syndrome and breast cancer, two common mechanisms are generally accepted. One mechanism lies in insulin resistance and hyperinsulinemia, which are especially associated with abdominal obesity, and appear to be central to the development of metabolic syndrome and might contribute to dyslipidemia and altered levels of circulating estrogens (32, 33, 53). The other mechanism lies in chronic inflammation caused by the accumulation of immune cells in adipose tissue and impaired secretion of adipokines, including a variety of proinflammatory cytokines, which could be a further linking factor between breast cancer and systemic insulin resistance (32). Clearly, adipokines secreted by adipose tissue are implicated in both mechanisms. Moreover, the present results for WC and the HW phenotype point to abnormalities in lipid metabolism. Contrary to the roles of most adipokines in proinflammation and carcinogenesis (54, 55), adiponectin—the most abundant adipokine—mainly exhibits inverse properties (56). Details of the signal pathway by which adiponectin acts have been summarized in several milestone reviews (38, 43, 56). In brief, the comprehensive role of adiponectin could be embodied within two pathways: first, by directly inhibiting breast cancer cell proliferation and promoting apoptosis; second, by acting on the receptors AdipoR1/R2, binding the APPL-1 protein, and stimulating the downstream pathway leading to insulin-sensitizing and anti-inflammatory effects, in turn, directly suppressing metabolic syndrome and indirectly suppressing antineoplastic properties.

Considering the known unique cellular and molecular mechanisms and scarce epidemiological data, along with the obtained results, we attempted to assess adiponectin as a potential mechanism. We confirmed the association between increased adiponectin and decreased metabolic syndrome regardless of menopausal status and form of adiponectin. Similarly, our previous study with the same project verified that HMW adiponectin was associated with decreased breast cancer risk, especially in postmenopausal women (39). What's more, we have found that when considering the influence of metabolic abnormality on the occurrence of breast cancer, the adiponectin showed a significant association with breast cancer only in the HW phenotype population. That is, adiponectin may function as one of the potential mechanisms linking metabolic abnormality and breast cancer because of its distinctive biological behaviors in all adipokines. In this regard, the contribution of adiponectin to breast cancer occurrence and progression is still controversial. Several studies found that adiponectin acted as a negative regulator of estrogen receptor alpha negative breast cancer, while adiponectin might restrain the development of estrogen receptor alpha positive breast cancer when at relatively low concentrations (57–60). The present study also found that adiponectin was associated with estrogen receptor-positive/progestogen receptor-positive and estrogen receptor-negative/progestogen receptor-negative breast cancer with the HW phenotype. Consistent with what we found in our previous study, namely, that general obesity, as indicated by BMI, was associated with the ER+/PR+ subtype, whereas central obesity, as indicated by waist/hip ratio, was more specific for the ER–/PR– subtype (61), We revealed that HW phenotype was an independent risk factor for the ER+/PR+ subtype. Therefore, the HW phenotype might function as a stronger marker of dysfunctional visceral lipid metabolism than BMI or WHR in predicting breast cancer risk. For physiologically adiponectin governed glucose levels and lipid metabolism (62), it might mediate the cross-talk between the HW phenotype and breast cancer especially subtyped by joint of ER and PR status.

In summary, our findings from the large Chinese representative data indicated that metabolic syndrome especially the HW phenotype, can significantly increase breast cancer risk, which was closely related to the “Western/new affluence” lifestyle, characterized by high energy intake and physical inactivity. Fortunately, healthy dietary patterns and an active lifestyle may play important roles in reducing the metabolic syndrome, which could be adopted as approaches for the prevention of breast cancer (63–65). Indeed, previous studies have shown an inverse relationship between metabolic syndrome and the Mediterranean diet, and metabolic syndrome could be reversed by adherence to the Mediterranean diet, with a reduction in the prevalence of metabolic syndrome to one-third after 2 years of the diet (66–68). Moreover, multiple studies have indicated that nutritional modifications and higher physical activity could attenuate the risk of breast cancer. The Iowa Women's Health Study confirmed that high levels of physical activity reduced the risk of post-menopausal breast cancer by 14% (65). Besides, one published study estimated that more than 30% of breast cancer cases could be prevented by lifestyle modification (65, 69). Therefore, as a result of the shift to “Western/new affluence” lifestyle, the rising prevalence of metabolic syndrome as well as breast cancer could be attenuated to some extent through lifestyle intervention and conservative therapy of underlying metabolic conditions. These days, much effort is being focused on encouraging lifestyle changes in adults.

Our study had several strengths. First, we further assessed the association between breast cancer risk and metabolic syndrome with special clusters of factors, and this study provided the first confirmation that the HW phenotype increased breast cancer risk. Second, we evaluated metabolic syndrome as an entity in its association with breast cancer among Chinese women across a wide geographic region (11 provinces) and using a relatively large sample. Third, by leveraging intact data, we performed the study by strictly following the diagnostic criteria rather than neglecting or replacing components. Fourth, the diagnostic criteria for metabolic syndrome are relatively recent. The 2009 Joint Statement and 2007 CDS definitions are first used in this study to evaluate the association of metabolic syndrome with breast cancer. Concurrently, our study also has several potential limitations. First, we only analyzed the data at baseline with no follow-up conducted, which could have provided a comprehensive evaluation of metabolic syndrome and breast cancer. Second, regarding molecule subtype age, we did not obtain results of metabolic syndrome with respect to breast cancer subtype. Third, due to the observational nature of the study, the precise mechanism for the results could not be fully explained, warranting further clarification. Despite these limitations, the study is meaningful in that it is the first retrospective study regarding this issue performed with a large Chinese population.

## Conclusions

To conclude, a large WC is strongly associated with increased breast cancer risk in premenopausal women. Metabolic syndrome with special cluster factors is related to breast cancer risk, and the HW phenotype significantly increases breast cancer risk. The rising prevalence of metabolic syndrome as well as breast cancer, which is attributable, at least partly, to the shift to the “Western/new affluence” lifestyle, could be attenuated to some extent through lifestyle intervention and conservative therapy of underlying metabolic conditions. As an important factor involved in fat metabolism, adiponectin, especially low adiponectin levels, may explain the association between metabolic abnormality and breast cancer to some extent. Further research into this mechanism and epidemiological studies are needed.

## Data Availability Statement

All datasets generated for this study are included in the article/Supplementary Material.

## Ethics Statement

The study protocols and procedures were approved by the Institutional Review Board at the Second Hospital of Shandong University. Written informed consent was obtained from each participant by investigators as part of the interview.

## Author Contributions

YX, WZ, and ZYu conceived and designed the experiments and wrote the paper. YX, WZ, ZYu, XD, ZF, SW, SL, FW, LY, FZ, LLi, QZ, QF, DG, SC, CG, XC, ZYa, XW, HL, HJ, HW, GL, QW, JZ, FJ, JT, FT, and CY performed the experiments. YX, WZ, and LLiu analyzed the data. SH and ZM contributed reagents, materials, analysis tools. ZYu supplied suggestion on study design as well as manuscript preparation.

### Conflict of Interest

The authors declare that the research was conducted in the absence of any commercial or financial relationships that could be construed as a potential conflict of interest.
