# Addendum: Metabolic Syndrome, and Particularly the Hypertriglyceridemic-Waist Phenotype, Increases Breast Cancer Risk, and Adiponectin Is a Potential Mechanism: A Case–Control Study in Chinese Women

**DOI:** 10.3389/fendo.2020.00227

**Published:** 2020-06-04

**Authors:** Yujuan Xiang, Wenzhong Zhou, Xuening Duan, Zhimin Fan, Shu Wang, Shuchen Liu, Liyuan Liu, Fei Wang, Lixiang Yu, Fei Zhou, Shuya Huang, Liang Li, Qiang Zhang, Qinye Fu, Zhongbing Ma, Dezong Gao, Shude Cui, Cuizhi Geng, Xuchen Cao, Zhenlin Yang, Xiang Wang, Hong Liang, Hongchuan Jiang, Haibo Wang, Guolou Li, Qitang Wang, Jianguo Zhang, Feng Jin, Jinhai Tang, Fuguo Tian, Chunmiao Ye, Zhigang Yu

**Affiliations:** ^1^Department of Breast Surgery, The Second Hospital of Shandong University, Jinan, China; ^2^Institute of Translational Medicine of Breast Disease Prevention and Treatment, Shandong University, Jinan, China; ^3^School of Medicine, Shandong University, Jinan, China; ^4^Breast Disease Center, Peking University First Hospital, Beijing, China; ^5^Department of Breast Surgery, The First Hospital of Jilin University, Changchun, China; ^6^Breast Disease Center, Peking University People's Hospital, Beijing, China; ^7^Department of Breast Surgery, Affiliated Tumor Hospital of Zhengzhou University, Zhengzhou, China; ^8^Breast Center, The Fourth Hospital of Hebei Medical University, Shijiazhuang, China; ^9^Department of Breast Surgery, Tianjin Medical University Cancer Institute and Hospital, Tianjin, China; ^10^Department of Thyroid and Breast Surgery, The First Affiliated Hospital of Binzhou Medical University, Binzhou, China; ^11^Department of Breast Surgery, Cancer Hospital, Chinese Academy of Medical Sciences, Beijing, China; ^12^Department of General Surgery, Linyi People's Hospital, Linyi, China; ^13^Department of General Surgery, Beijing Chaoyang Hospital, Beijing, China; ^14^Breast Center, Qingdao University Affiliated Hospital, Qingdao, China; ^15^Department of Breast and Thyroid Surgery, Weifang Traditional Chinese Hospital, Weifang, China; ^16^Department of Breast Surgery, The Second Affiliated Hospital of Qingdao Medical College, Qingdao Central Hospital, Qingdao, China; ^17^Department of General Surgery, The Second Affiliated Hospital of Harbin Medical University, Harbin, China; ^18^Department of Breast Surgery, The First Affiliated Hospital of China Medical University, Shenyang, China; ^19^Department of General Surgery, Nanjing Medical University Affiliated Cancer Hospital Cancer Institute of Jiangsu Province, Nanjing, China; ^20^Department of Breast Surgery, Shanxi Cancer Hospital, Taiyuan, China; ^21^Suzhou Institute, Shandong University, Suzhou, China

**Keywords:** breast cancer, metabolic syndrome, hypertriglyceridemic-waist phenotype, adiponectin, risk

In the original article, there were mistakes in Table 6, Table 7, Table 9 as published. The numbers of patients in Table 6 and **Table 9** were incorrect. The contents in Table 7 and Table 9 were repetitive to some degree in that we had shown the association between adiponectin with metabolic syndrome and HW phenotype. Therefore, for this Correction, we analyzed the association between adiponectin and metabolic syndrome, and the association in pre- and postmenopausal subgroups in Table 7. In Table 9, we converted the numerical variable into categorical variable, which should provide better guide for clinical practice. In our view, this avoids the repetition. These new tables appear below as Tables 6, 7, 9. The authors apologize for these errors and any confusion that may have arisen due to them and hopes these additional tables sufficiently addresses them.

**Table 6:** Association between HW phenotype and breast cancer by logistic regression.

	**All subjects(*****n*** **=** **595)**			**Premenopausal(*****n*** **=** **383)**			**Postmenopausal(*****n*** **=** **209)**			**ER+/PR+(*****n*** **=** **293)**			**ER+/PR–(*****n*** **=** **59)**			**ER–/PR–(*****n*** **=** **148)**		
	**OR**	**95% CI**	***P***	**OR**	**95% CI**	***P***	**OR**	**95% CI**	***P***	**OR**	**95% CI**	***P***	**OR**	**95% CI**	***P***	**OR**	**95% CI**	***P***
**Univariate model**																		
WC+TG (normal = reference)	1.66	1.10–2.50	0.016	1.63	0.95–2.82	0.077	1.72	0.91–3.22	0.09	2.06	1.29–3.27	0.002	1.70	0.73–4.00	0.222	1.43	0.76–2.67	0.266
**Multivariate model^a^**																		
WC+TG (normal=reference)	1.56	1.02–2.39	0.039	1.49	0.85–2.63	0.167	1.60	0.82–3.12	0.170	1.95	1.21–3.13	0.006	1.71	0.72–4.08	0.225	1.21	0.63–2.33	0.571

*WC, waist circumference; TG, triglycerides; ER, estrogen receptor; PR, progesterone receptor*.

a *Adjusted for age, number of childbirths, age at menarche, breastfeeding, smoking, alcohol use, family history of breast cancer, and contraceptive drug use*.

**Table 7:** Association between total adiponectin, HMW adiponectin, HMW/total ratio, and metabolic syndrome.

	**All subjects**			**Premenopausal**			**Postmenopausal**		
	**With MetS**	**Without MetS**	***p***	**With MetS**	**Without MetS**	***p***	**With MetS**	**Without MetS**	***p***
Total adiponectin	5.970 ± 3.789	2.807 ± 2.007	0.004	5.960 ± 3.830	6.637 ± 3.558	0.054	5.979 ± 3.762	6.909 ± 3.875	0.022
HMW adiponectin	2.408 ± 1.870	2.807 ± 2.007	0.004	2.371 ± 1.830	2.757 ± 1.958	0.037	2.445 ± 1.915	2.935 ± 2.116	0.024
HMW/total ratio	0.39 ± 0.14	0.41 ± 0.16	0.101	0.39 ± 0.14	0.40 ± 0.17	0.233	0.39 ± 0.15	0.42 ± 0.15	0.150

*MetS, metabolic syndrome; HMW, high molecular weight*.

**Table 9:** The association among metabolic syndrome, breast cancer, and adiponectin.

		**Controls**	**All cases**		**ER+/PR+**		**ER+/PR–**		**ER–/PR–**	
**METABOLIC SYNDROME**										
YES										
	Total adiponectin			0.362		0.944		0.764		0.203
	High	26 (22.2%)	27 (17.8%)		17 (21.8%)		3 (15.8%)		5 (12.8%)	
	Low	91 (77.8%)	125 (82.2%)		61 (78.2%)		16 (84.2%)		34 (87.2%)	
	HMW adiponectin			0.296		0.597		0.113		0.403
	High	66 (56.4%)	76 (50.0%)		41 (52.6%)		7 (36.8%)		19 (48.7%)	
	Low	51 (43.6%)	76 (50.0%)		37 (47.4%)		12 (63.2%)		20 (51.3%)	
	HMW/total ratio			0.354		0.069		0.805		0.711
	High	59 (50.4%)	68 (44.7%)		29 (37.2%)		9 (47.4%)		21 (53.8%)	
	Low	58 (49.6%)	84 (55.3%)		49 (62.8%)		10 (52.6%)		18 (46.2%)	
No										
	Total adiponectin			0.097		0.121		0.339		0.118
	High	106 (25.5%)	92 (20.8%)		43 (20.0%)		13 (32.5%)		20 (18.3%)	
	Low	309 (74.5%)	351 (79.2%)		172 (80.0%)		27 (67.5%)		89 (81.7%)	
	HMW adiponectin			0.507		0.970		0.588		0.244
	High	287 (69.2%)	297 (67.0%)		149 (69.3%)		26 (65.0%)		69 (63.3%)	
	Low	128 (30.8%)	146 (33.0%)		66 (30.7%)		14 (35.0%)		40 (36.7%)	
	HMW/total ratio			0.359		0.229		0.873		0.062
	High	213 (51.3%)	213 (48.2%)		99 (46.3%)		20 (50.0%)		45 (41.3%)	
	Low	202 (48.7%)	229 (51.8%)		115 (53.7%)		20 (50.0%)		64 (58.7%)	
**HW PHENOTYPE**										
YES										
	Total adiponectin			0.005		0.028		1.000		0.043
	High	14 (35.9%)	9 (13.0%)		6 (14.6%)		2 (28.6%)		1 (6.7%)	
	Low	25 (64.1%)	60 (87.0%)		35 (85.4%)		5 (71.4%)		14 (93.3%)	
	HMW adiponectin			0.717		0.527		0.424		0.583
	High	24 (61.5%)	40 (58.0%)		28 (68.3%)		3 (42.9%)		8 (53.3%)	
	Low	15 (38.5%)	29 (42.0%)		13 (31.7%)		4 (57.1%)		7 (46.7%)	
	HMW/total ratio			0.570		0.263		1.000		0.839
	High	17 (43.6%)	34 (49.3%)		23 (56.1%)		3 (42.9%)		7 (46.7%)	
	Low	22 (56.4%)	35 (50.7%)		18 (43.9%)		4 (57.1%)		8 (53.3%)	
NO										
	Total adiponectin			0.247		0.442		0.632		0.150
	High	118 (23.9%)	110 (20.9%)		54 (21.4%)		14 (26.9%)		24 (18.0%)	
	Low	375 (76.1%)	416 (79.1%)		198 (78.6%)		38 (73.1%)		109 (82.0%)	
	HMW adiponectin			0.252		0.505		0.191		0.157
	High	329 (66.7%)	333 (63.3%)		162 (64.3%)		30 (57.7%)		80 (60.2%)	
	Low	164 (33.3%)	193 (36.7%)		90 (35.7%)		22 (42.3%)		53 (39.8%)	
	HMW/total ratio			0.136		0.011		0.813		0.132
	High	255 (51.7%)	247 (47.0%)		105 (41.8%)		26 (50.0%)		59 (44.4%)	
	Low	238 (48.3%)	278 (53.0%)		146 (58.2%)		26 (50.0%)		74 (55.6%)	

*ER, estrogen receptor; PR, progesterone receptor*.

*Cut-off value of high and low level for total adiponectin, HMW adiponectin, and HMW/total ratio is 8.768, 1.635, and 0.399, respectively*.

In the original article, corresponding text of Table 6, Table 7, and Table 9 was corrected.

A correction has been made to Abstract, Results, Paragraph number 1:

In addition, total adiponectin levels among breast cancer patients were much lower than among controls (*p* = 0.005) only in the HW phenotype subgroup. Furthermore, the HW phenotype was associated with increased risk of estrogen receptor/progesterone receptor-positive (ER+/PR+) breast cancer, with a 95% (OR = 1.95, 95% CI:1.21–3.13) increase. However, there was no significant association between the HW phenotype and both ER+/PR– and ER–/PR– subtypes.

A correction has been made to Results, Cluster Mode of HW Phenotype Significantly Increases Breast Cancer Risk, Paragraph number 3:

HW phenotype was associated with ER+/PR+ breast cancer, with a 95% (OR = 1.95, 95% CI:1.21–3.13) increase in risk for women with a positive HW phenotype. However, there was no significant association between HW phenotype and both ER+/PR– and ER–/PR– subtypes.

A correction has been made to Results, Adiponectin Might Be the Mechanism Linking Metabolic Syndrome to Breast Cancer, Paragraph number 2:

total adiponectin levels among breast cancer patients were much lower than among the controls(*p* = 0.005) in the HW phenotype subgroup.

A correction has been made to Results, Adiponectin Might Be the Mechanism Linking Metabolic Syndrome to Breast Cancer, Paragraph number 3:

there was a significant difference of total adiponectin in ER+/PR+ (*p* = 0.028) and ER–/PR– (*p* = 0.043) breast cancer compared to the controls, who were much lower in the HW phenotype subgroup.

A correction has been made to Discussion, Paragraph number 6:

We revealed that HW phenotype was an independent risk factor for the ER+/PR+ subtype.

The authors apologize for this error and state that this does not change the scientific conclusions of the article in any way. The original article has been updated.

