# Supplementary material for: Metabolic Syndrome, and Particularly the Hypertriglyceridemic-Waist Phenotype, Increases Breast Cancer Risk, and Adiponectin Is a Potential Mechanism: A Case–Control Study in Chinese Women
Source: Front Endocrinol (Lausanne). 2020 Jan 21;10:905. doi: 10.3389/fendo.2019.00905 (PMC6990117; doi:10.3389/fendo.2019.00905)
Supplement: Supplementary file 1 [file Table_1.DOCX]

| Supplemental Table 1. Criteria for metabolic syndrome | | | | |
| --- | --- | --- | --- | --- |
|  | ATPIII(2005) | IDF(2005) | CDS(2007) | Joint Statement(2009) |
| Elevated waist circumference | ≥ 80 cm | ≥ 80 cm | > 85 cm | ≥ 80 cm |
| Reduced HDL | <1.3 mmol/L or  drug treatment for reduced HDL | < 1.29 mmol/l or  specific treatment for this lipid abnormality | <1.04mmol/L | <1.3 mmol/L or  drug treatment for reduced HDL |
| Elevated triglycerides | ≥ 1.7mmol/Lor  drug treatment for elevated triglycerides | 1.7 mmol/l or  specific treatment for lipid abnormality | ≥ 1.7 mmol/l | ≥ 1.7mmol/L or  drug treatment for elevated triglycerides |
| Elevated blood pressure | ≥130 mm Hg systolic blood pressure or  ≥85 mm Hg diastolic blood pressure or  On antihypertensive drug treatment in a patient with a history of hypertension | Systolic: 130 mmHg  or  Diastolic: 85 mmHg  or  treatment of previously diagnosed hypertension | ≥130/85 mmHg  or treatment of previously diagnosed hypertension; | ≥130 mm Hg systolic blood pressure  or ≥85 mm Hg diastolic blood pressure or  On antihypertensive drug treatment in a patient with a history of hypertension |
| Elevated fasting glucose | ≥5.6 mmol/L or  On drug treatment for elevated glucose | Fasting plasma glucose 5.6 mmol/l or  previously diagnosed Type 2 diabetes | Fasting plasma glucose 6.1 mmol/l or 2h postprandial plasma glucose 7.8mmol/l, or previously diagnosed type 2 diabetes | ≥5.6 mmol/L  or On drug treatment for elevated glucose |
| Criterion | Any 3 of 5 constitute diagnosis of metabolic syndrome | Central obesity plus any 2 of 4 others constitute diagnosis of metabolic syndrome | Any 3 of 5 constitute diagnosis of metabolic syndrome | Any 3 of 5 constitute diagnosis of metabolic syndrome |
